# Inter-rater variability of three-dimensional fracture reduction planning according to the educational background

**DOI:** 10.1186/s13018-021-02312-w

**Published:** 2021-02-25

**Authors:** Christoph Zindel, Philipp Fürnstahl, Armando Hoch, Tobias Götschi, Andreas Schweizer, Ladislav Nagy, Simon Roner

**Affiliations:** 1 Research in Orthopedic Computer Science (ROCS), Balgrist University Hospital, University of Zurich, Balgrist CAMPUS, Zurich, Switzerland; 2 Department of Orthopedics, Balgrist University Hospital, University of Zurich, Zurich, Switzerland; 3 Institute for Biomechanics, ETH Zurich, Zurich, Switzerland

**Keywords:** Distal radius fracture, Computer-assisted planning, Fracture reduction, Three-dimensional displacement analysis, Planning experience

## Abstract

**Background:**

Computer-assisted three-dimensional (3D) planning is increasingly delegated to biomedical engineers. So far, the described fracture reduction approaches rely strongly on the performance of the users. The goal of our study was to analyze the influence of the two different professional backgrounds (technical and medical) and skill levels regarding the reliability of the proposed planning method. Finally, a new fragment displacement measurement method was introduced due to the lack of consistent methods in the literature.

**Methods:**

3D bone models of 20 distal radius fractures were presented to nine raters with different educational backgrounds (medical and technical) and various levels of experience in 3D operation planning (0 to 10 years) and clinical experience (1.5 to 24 years). Each rater was asked to perform the fracture reduction on 3D planning software.

**Results:**

No difference was demonstrated in reduction accuracy regarding rotational (*p* = 1.000) and translational (*p* = 0.263) misalignment of the fragments between biomedical engineers and senior orthopedic residents. However, a significantly more accurate planning was performed in these two groups compared with junior orthopedic residents with less clinical experience and no 3D planning experience (*p* < 0.05).

**Conclusion:**

Experience in 3D operation planning and clinical experience are relevant factors to plan an intra-articular fragment reduction of the distal radius. However, no difference was observed regarding the educational background (medical vs. technical) between biomedical engineers and senior orthopedic residents. Therefore, our results support the further development of computer-assisted surgery planning by biomedical engineers. Additionally, the introduced fragment displacement measure proves to be a feasible and reliable method.

**Level of Evidence:**

Diagnostic Level II

## Background

The restoration of the joint surface by anatomical reduction is the primary goal in the surgical treatment of intra-articular distal radius fractures [1–5]. Through preoperative planning, a better understanding of the fracture pathology, the order of reduction of multiple fragments, and consequently, a more accurate choice of the surgical approach or a better choice of the implant could be eventually achieved [6]. Preoperative planning is commonly performed by surgeons on two-dimensional (2D) images from plain radiographs [6]. However, 2D preoperative planning often provides insufficient information to understand the three-dimensional (3D) complexity of the fracture morphology [7–11]. Due to recent developments in computer-aided design (CAD) software and rapid prototyping technology, accurate 3D preoperative simulations became widely accessible [12–16]. So far, 3D preoperative simulations are applied in the planning of osteotomies and show advantages over plain radiographs or 2D computed tomography (CT) in visualization and quantification of rotational malunions and intra-articular steps and gaps [17–22]. The biggest advantage of 3D preoperative simulation in fractures is the possibility to gain an exact understanding of all fracture lines and surfaces and to simulate the reduction of all fragments. Different approaches are described in the literature and used in daily life for simulating fracture reduction: (1) free-hand visual alignment, (2) the incorporation of the mirrored contralateral side to facilitate reduction [23–27], (3) the use of a statistical shape model (SSM) [28], or (4) attempts to use automatic alignment algorithms [29]. None of these methods is fully automated or yet applicable in the clinical use and hence all rely strongly on the performance of the users itself, which could influence treatment decisions and outcome. Influencing factors can reach from surgical, respectively anatomical, knowledge, the amount of practice in using 3D programs as far as to experience in 3D operation planning itself [23–28]. In general, surgeons lack training in 3D planning, and in our experience, few training opportunities are available to them. Also because of the technical complexity, the 3D planning is increasingly transferred to biomedical engineers. This way, the technical expertise becomes easily available for the (not technology affine) surgeons but thereof depend on a close cooperation between them and the engineer to transfer medical knowledge.

However, it is still unclear if enough medical, in particular anatomical, knowledge is existent in this rather new subgroup of biomedical engineers for independent 3D planning. Therefore, we investigated the difference in fracture reduction accuracy regarding the overall experience in 3D planning of the user (in years) as well as the educational background (medical versus technical). We hypothesize that trained biomedical engineers have enough anatomical knowledge to perform 3D fracture reduction and perform no worse than surgeons in training. To be able to measure the variability between different raters in a standardized and automatic way, we developed a method for validating the displacement of fragments in 3D.

## Methods

In this retrospective study, we included CT data of 20 patients with a distal radius fracture acquired between August 2016 and September 2017. The age range was 15 to 70 years with a mean age of 41.9 years (SD 15.5). The included radii were 10 times a right radius and 10 times a left radius. The 20 fractures were classified using the AO/OTA classification system (2× 2R3A, 4× 2R3B, 14× 2R3C) [30]. Each of the 20 fractures consisted of 1 to 6 fragments (mean number of fragments = 3.15), from which only the intra-articular fragments were included in the study. Overall, 51 fragments from 20 different cases of distal radius fractures (an average of 2.55 fragments per case) were included in the evaluation. More detailed information of the demographics is provided in Table 1.

**Table 1:** Demographics of the subjects

Patient	Age (years)	Sex	Side	Total number of fragments	Number of fragments included in statistic	AO classification
1	31	m	R	4	3	2R3C
2	60	m	L	4	3	2R3C
3	46	f	L	7	6	2R3C
4	53	f	L	2	2	2R3C
5	34	f	R	3	3	2R3C
6	29	f	L	1	1	2R3A
7	20	f	R	3	2	2R3C
8	15	f	R	3	2	2R3C
9	48	f	L	3	2	2R3C
10	50	f	R	2	1	2R3A
11	28	m	L	2	2	2R3B
12	67	m	R	3	2	2R3C
13	45	m	R	2	2	2R3B
14	40	m	L	2	2	2R3B
15	23	f	R	1	1	2R3B
16	23	f	L	3	2	2R3C
17	55	m	L	7	4	2R3C
18	66	m	R	3	3	2R3C
19	51	m	R	3	3	2R3C
20	51	m	L	5	5	2R3C
**Avg.**	**41.9**	**10× m** **10× f**	**10× R 10× L**	**3.15**	**2.55**	**2× 2R3A** **4× 2R3B** **14× 2R3C**

*m* male, *f* female, *R* right, *L* left

**Fig. 1:**
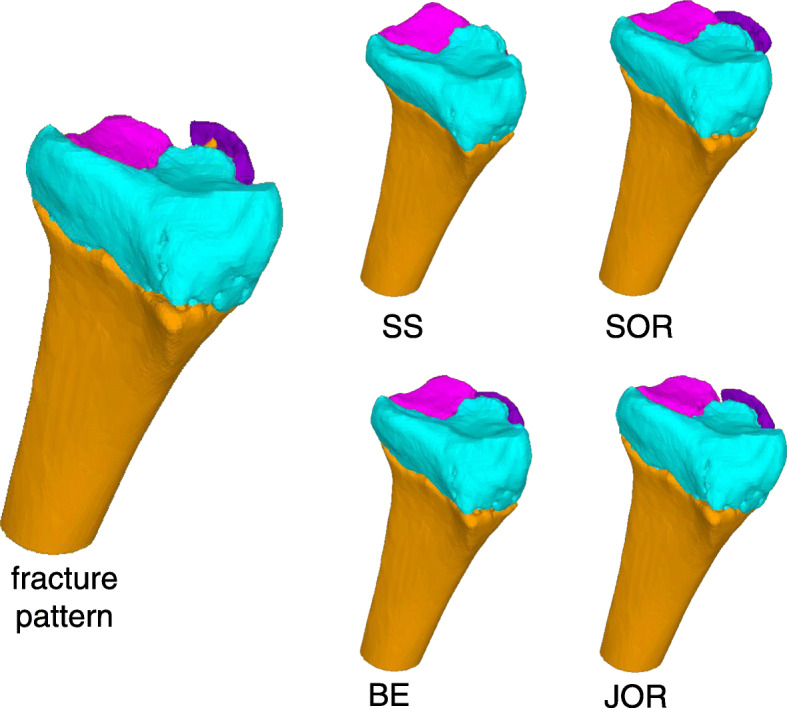
Initial position of the fracture pattern and an example of each group of the resulting reduction plans: senior surgeon (SS), biomedical engineer (BE), senior orthopedic resident (SOR), and junior orthopedic resident (JOR). The dorsal flake fragment (purple) was not included in the analysis

**Fig. 2:**
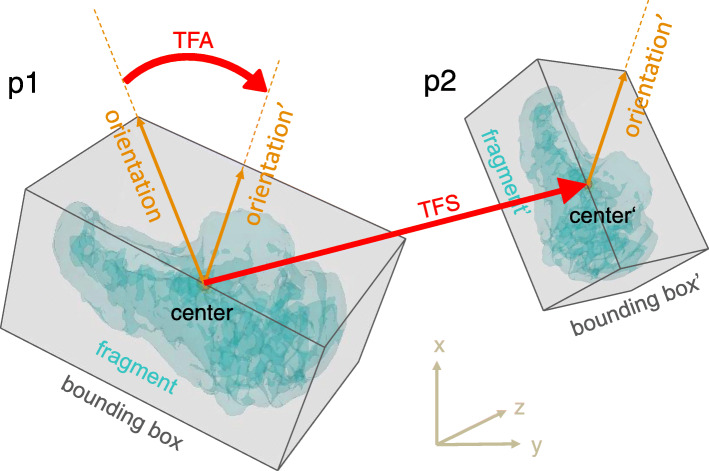
Calculation of the fragment transformation (transformation shift (TFS) and transformation angle (TFA)). A translocated fragment is shown in its initial position p1 and in its new transformed position p2

The image data had been acquired using a CT device (Siemens SOMATOM Definition AS, Siemens Healthcare, Erlangen, Germany) with a slice thickness of 1.0 mm (120 kV). 3D bone models were extracted from the CT data with a commercial segmentation software (Mimics 19.0; Materialise NV, Leuven, Belgium) using thresholding, region growing, and the marching cubes algorithm as described before [31]. Each bone fragment of the fracture was segmented separately to 3D bone models and imported into a preoperative planning software (CASPA, CARD AG, Zurich, Switzerland). Nine raters were selected according to their profession, clinical experience, and 3D operation planning experience, including fractures and osteotomies of hand and forearm bones, as shown in Table 2. The clinical experience was interpreted as a measure for medical, in particular anatomical, knowledge of orthopedic surgeons. The raters included seven medical doctors and two biomedical engineers (BE). Three medical doctors were junior orthopedic residents (JOR), three medical doctors were senior orthopedic residents (SOR), and one was a senior orthopedic surgeon (SS). The medical doctors had clinical experience in a range of 1.5 up to 24 years and from marginal (< 1 year) experience in 3D operation planning up to 10 years. The senior orthopedic surgeon (SS) was defined as the gold standard with its 24 years of clinical and 10 years of 3D planning experience. The biomedical engineers had 2, respectively 3 years of experience in 3D operation planning. Each rater was familiar to the planning software due to previous work. All raters were introduced to the study goal and to the reduction task equally. The reduction task comprised the simulation of the reduction of each 3D bone model from the initial position to an optimal anatomical alignment with articular congruency. Fracture reduction was performed by interactive displacement and rotation of the fragments with the computer mouse from multiple viewpoints. The viewpoints were freely defined by the raters with the mouse. There was no constraint of time nor a restriction in the order of placing the fragments. Figure 1 shows a bone model in the initial position and the different reduction plans of one rater per group. The reduction plans of all raters were then compared with the plan of the SS (gold standard). To calculate the difference of the reduction plans, we introduced a new fragment displacement measure. The proposed fragment displacement measure permits standardized measurements in a completely automatic procedure. The 3D displacement of each fragment is represented by only two parameters: a pure 3D shift and a pure 3D rotation. To describe the displacement of a fragment from position p1 (pre-reduction) to position p2 (post-reduction), a center point of the fragment has to be calculated in a reproducible, automated way. A so-called oriented bounding box [32] was automatically calculated, which is the uniquely defined box with minimal volume covering the fragment. The center of this bounding box was defined as the center of the fragment. The transformation shift (TFS) was then calculated as the length of the 3D displacement vector from the center point of the fragment in pre-reduced position to the center point of the fragment in post-reduced position. The second value, the transformation angle (TFA), was defined as the pure rotational difference of a fragment from pre- to post-reduction position by angle φ around the center point. This angle is calculated automatically by using quaternions derived from the Horn transformation [33]. TFS and TFA are independent variables. Figure 2 shows an example of the proposed fragment displacement measure.

**Table 2:** Overview of raters

Rater	Profession	Clinical experience (years)	3D planning experience (years)
1	SS	24	10
2	BE	-	2.0
3	BE	-	3.0
4	SOR	6.0	1.5
5	SOR	6.0	1.0
6	SOR	7.0	0.0
7	JOR	1.5	0.0
8	JOR	3.0	0.0
9	JOR	1.5	0.0

*SS* senior surgeon, *BE* biomedical engineer, *SOR* senior orthopedic resident, *JOR* junior orthopedic resident

### Statistical analysis

Deviations from the gold standard planning of each bone fragment were averaged per rater and case (*n* = 20). These average planning differences were further aggregated to yield an average difference per rater (*n* = 8) and the standard deviation thereof. These two parameters (TFA and TFA) were statistically analyzed separately with the intention to represent the rater’s average performance and consistency, respectively. The effect of profession, clinical, and 3D planning experience was assessed with an ANOVA and subsequent Bonferroni-corrected post hoc tests for the first, and with a linear regression model for the latter two factors. *p* values < 0.05 were considered statistically significant. Statistical analysis was performed with SPSS (IBM SPSS Statistics for Windows, Version 26.0. Armonk, NY: IBM Corp.).

## Results

**Fig. 3:**
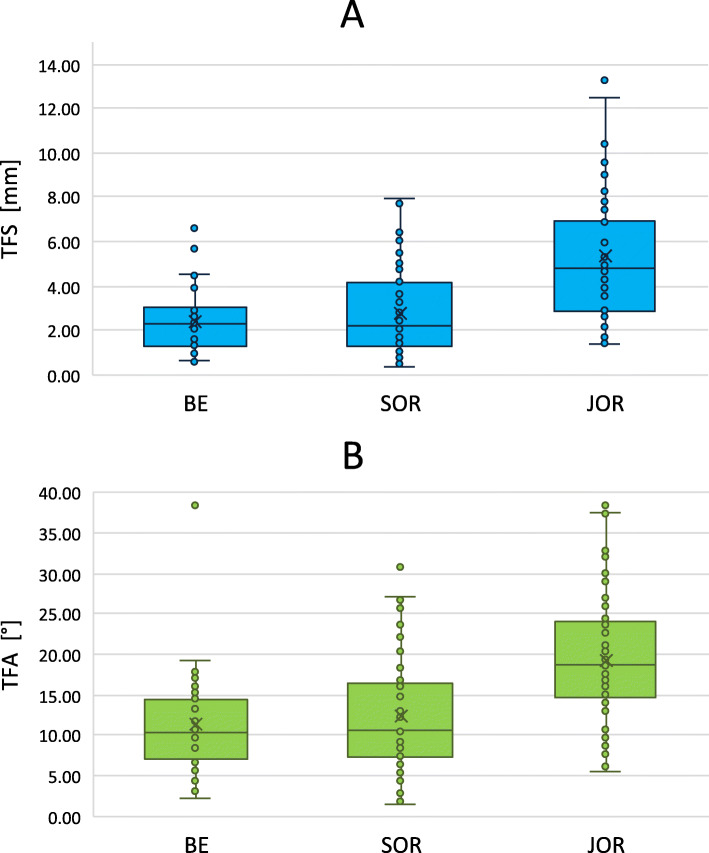
Distribution of the transformation shift (TFS) and the transformation angle (TFA) per group. **a** shows the distribution of the TFS and **b** shows the distribution of the TFA according to the performance of the 3 rater groups: biomedical engineer (BE), senior orthopedic resident (SOR), and junior orthopedic resident (JOR)

**Fig. 4:**
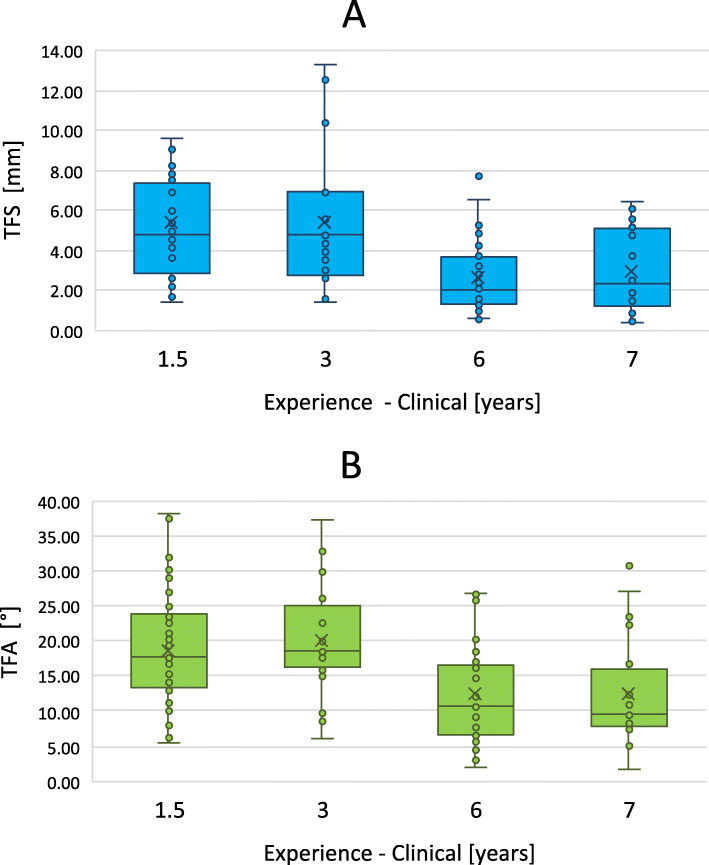
**a** shows the distribution of the transformation shift (TFS) and **b** shows the distribution of the Transformation-Angle (TFA) according to the clinical experience of the raters

**Fig. 5:**
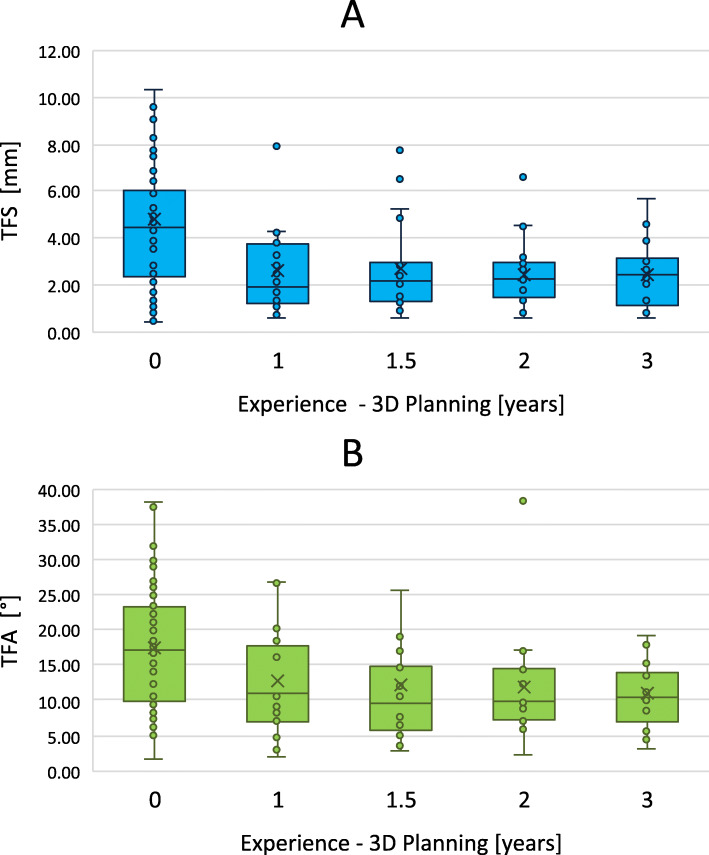
**a** shows the distribution of the transformation shift (TFS) and **b** shows the distribution of the transformation angle (TFA) according to the experience in 3D planning of the raters

The distribution of the TFS per rater are illustrated in Fig. 3a. The performances among the three profession groups differed significantly (F(2, 7) = 296.686, *p* < 0.01). Post hoc analyses revealed a significant better fragment reduction of SOR (*p* < 0.01) and BE (*p* < 0.01) than JOR. In contrast, the difference between SOR and BE was not significantly different (*p* = 0.263). The analysis of the consistency of TFS (standard deviation) showed a significant difference between the profession groups (F(2, 7) = 6.208, *p* = 0.044). The post hoc analyses revealed no significant difference between specific groups. The linear regression models show a significant influence of the experience in 3D planning (F(1, 6) = 7.515, *p* = 0.034), with a *R*^2^ of 0.556) as well as a significant influence of the clinical experience (F(1, 4) = 31.282, *p* < 0.01, with a *R*^2^ of 0.887) to the reduction performance. The analysis shows an improvement of the TFS by 0.943 mm per year of experience in 3D planning and by 0.560 mm per year of clinical experience. More details can be seen in Figs. 4a and 5a.

The distribution of the TFA per rater are illustrated in Fig. 3b. The TFA among the three profession groups differs significantly (F(2, 7) = 17.795, *p* < 0.01). Post hoc analyses show a significant better fragment reduction of SOR (*p* = 0.013) and BE (*p* = 0.011) than JOR. In contrast, the difference between SOR and BE was not significantly different (*p* = 1.000). The analysis of the consistency of TFA (standard deviation) showed no significant difference between the profession groups (F(2, 7) = 1.688, *p* = 0.275). The linear regression models show a significant influence of the experience in 3D planning (F(1, 6)= 6.216, *p* = 0.047), with a *R*^2^ of 0.509) as well as a significant influence of the clinical experience (F(1, 4) = 11.066, *p* = 0.029, with a *R*^2^ of 0.735) to the reduction performance. The analysis shows an improvement of the TFA by 2.472° per year of experience in 3D planning and by 1.394° per year of clinical experience. More details can be seen in Figs. 4b and 5b.

## Discussion

3D preoperative planning with its advantages over 2D planning becomes more and more adopted in orthopedic surgery. However, preoperative 3D fracture reduction is currently performed manually by surgeons or engineers due to the lack of clinical-ready automated algorithms. Even though some of the computer-assisted reduction methods give the impression to be standardized and reproducible, all algorithms described in the literature [23–28] strongly rely on medical, in particular anatomical, knowledge of the user. In this study, we investigated whether trained biomedical engineers can accurately plan reductions of distal radius fractures compared with resident orthopedic surgeons with various clinical and 3D planning experience.

Our results demonstrate that the experience of 3D planning and clinical experience of the users are relevant factors for the performance of preoperative fracture reduction planning. The most important finding is that we observed no difference in the planning accuracy between senior orthopedic residents and biomedical engineers, neither in TFS nor in TFA, but a significant difference to junior orthopedic residents in both variables. This outcome is surprising, considering the very different medical knowledge (measured in years of clinical experience) and educational background, but emphasizes this new profession in computer-assisted surgical methods. The analyses of the consistency of the performances by TFA show no difference between the three groups, which underlines the abovementioned finding as well. The consistency of the performance by TFA shows a significant difference between the groups, but no difference in the post hoc analyses, which does not allow further interpretation.

A prerequisite for measuring variability between different raters is a standardized and objective measurement method. So far, little data exists about reliable and validated displacement or transformation measures, which can be applied for 3D preoperative planning in orthopedic surgery [34, 35]. We developed such a method particularly for outcome analysis of the preoperative operation planning, but this method can also be used in analysis of navigation accuracy of computer-assisted surgeries. Compared with the other in the literature describing 3D displacement measurements [18, 22, 25, 36–39], our measurement method has several advantages. It is a mathematical method including coordinate system independence and, consequently, it is user-independent. The results are reproducible which allows a better comparison between future studies. Both measures, TFS and TFA, are statistically independent and thus, the results can easily be processed in statistics. Finally, the measures are intuitive and enable user-friendly use in the clinical routine.

A limitation of the present study is the small numbers of raters and the relatively small sample size of 20 planning cases. Therefore, an analysis of the reliability of the performance of the raters within the different groups, as for example an ICC analysis, could not be realized. This would be very interesting for the further investigation of preoperative 3D planning accuracy. Another limitation in the evaluation is that the time used for preoperative reduction planning was not recorded, which in our opinion is not relevant as there are few indications where a distal radius fracture needs to be operated on within a few hours (e.g., open fractures, neurological deficits, ...). In an unpublished work, we already demonstrated the feasibility of 3D planning and navigation by patient-specific instruments (PSI) for the treatment of distal radius fractures. The entire process from acquiring the CT image data until being able to perform the navigated surgery required comfortably 2–3 days. A technical shortcoming of current 3D preoperative reduction planning methods is the absence of cartilage models in order to include articular congruity in the reduction task. Moreover, only the reduction of the bone fragments was assessed, without further clinical knowledge such as the surgical approach and biomechanical considerations such as of ligaments. Finally, it would be interesting to investigate the accuracy of the reduction plans compared with a fully automated fragment reduction method. A further interest persists in the postoperative outcome (radiological and clinical) dependent on the method of preoperative planning.

## Conclusions

In conclusion, we found that experience in 3D operation planning and clinical experience are relevant factors to accurately plan an intra-articular reduction of the distal radius. No difference regarding the educational background (medical vs. technical) was observed and therefore supports the further development of computer-assisted surgical planning by biomedical engineers. The hereby used fragment displacement measure is a basic and easy tool to compare fragment transformation in 3D bone models. Consequently, we suggest using more standardized measurement methods for all the future work for comparison of fragment transformations in 3D bone models in order to make future studies more comparable.

## Data Availability

The datasets used and/or analyzed during the current study are available from the corresponding author on reasonable request.

## Abbreviations

2D: Two-dimensional
3D: Three-dimensional
BE: Biomedical engineers
CAD: Computer-aided design
CT: Computed tomography
JOR: Junior orthopedic residents
SOR: Senior orthopedic residents
SS: Senior orthopedic surgeon
SSM: Statistical shape model
TFA: Transformation angle
TFS: Transformation shift
